# Osteoradionecrosis in Subaxial Cervical Spine - a Rare and Devastating Complication: A Case Report

**DOI:** 10.5704/MOJ.1711.005

**Published:** 2017-11

**Authors:** MZ Rashid, MH Ariffin, SA Rhani, A Baharudin, K Ibrahim

**Affiliations:** Department of Orthopaedics, Universiti Kebangsaan Malaysia, Cheras, Malaysia

**Keywords:** osteoradionecrosis, subaxial, cervical, spine, hyperbaric oxygen therapy

## Abstract

Osteoradionecrosis, a rare complication of radiation therapy, is a slow progression disease which affects the surrounding structures of spinal components. It essentially weakens the soft tissue and bony configuration and can cause nerve impingement or cord compression. We describe a patient who underwent radiotherapy for thyroid cancer and presented with cervical kyphosis with anterolisthesis of C3/C4 and C4/C5 some 32 years later. We explore the role of anterior and posterior fusion, as well as hyperbaric oxygen therapy in promoting healing.

## Introduction

Osteoradionecrosis (ORN) is an entity affecting bone and its surrounding soft tissues and is widely known as a late complication of radiotherapy. It presents a daunting task to surgeons not only to correct the resultant spinal deformity in a defective bone, but also associated with poor healing, high risk of infection and problem with bony unions in an irradiated bed. Osteoradionecrosis was typically reported 4-9 years after nasopharyngeal carcinoma treatment^1^. It usually involves C1/C2 with resultant atlantoaxial instability. However, sub-axial cervical spine ORN is much rarer with limited literatures describing the occurrence and its treatment. We present a case of osteoradionecrosis of C4/C5 post-total thyroidectomy and radiotherapy for follicular thyroid cancer.

## Case Report

**Fig. 1:**
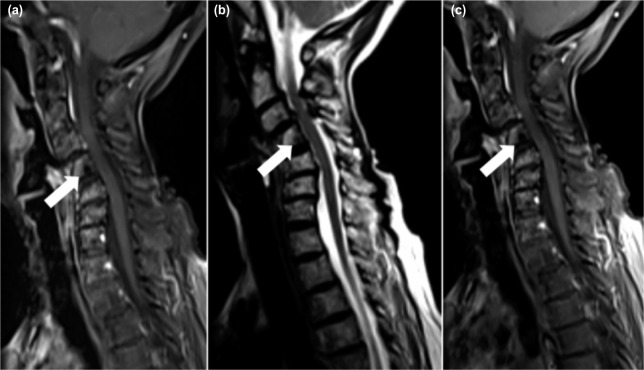
(a) T1FS image and (b) T2WI showing hypo intense area at posterior vertebral body C4. (c) Post contrast enhanced image (Gadolinum) showing non-enhancement over similar area of C4. Low intensity on all signals indicate vacuum cleft sign.

**Fig. 2:**
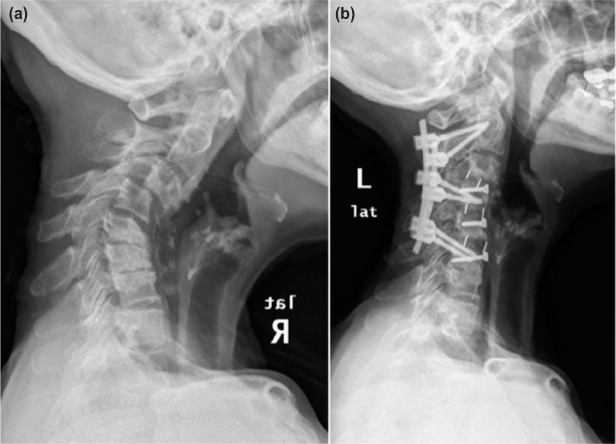
(a) Pre-operative and (b) one year post-operative lateral view of cervical spine. Cortical screws are used to buttress the intervertebral disc cage. Fusions of intervertebral bodies are seen.

**Fig. 3:**
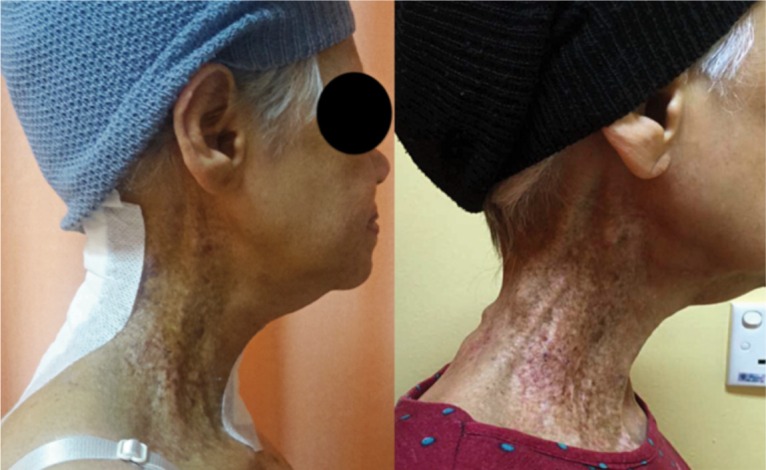
Postoperative clinical pictures showing maintenance of lordosis one year postoperative compared to immediate post-operative. Hyperpigmented skin over right-side post irradiation therapy.

We report the case of a 64-year old Malay lady with past history of follicular thyroid cancer 32 years ago treated with total thyroidectomy and radiotherapy. She presented with severe neck pain and radiculopathy to the left upper limb. Clinically, post-irradiation scarring and muscle wasting on right anterior triangle of her neck and left upper limb was noted, with reduction in power and sensation at C5C6 and brisk reflexes in all limbs. Radiographs showed an area of lucency at C4 anterior-inferior body and C5 at superior portion and cervical kyphosis measuring 62 degrees by Cobb method with anterolisthesis of more than 50% at C3/C4 and 25% at C4/C5 level. Stress dynamic radiography was not attempted for fear of worsening the neurologic deficit in the presence of gross radiological instability. Magnetic resonance imaging (MRI) in rigid cervical collar showed well defined hypo-intense area at C4 on T1 fat suppressed, T2 weighted image and non-enhancement post gadolinium contrast (Fig. 1). Patient underwent surgery in May 2016, utilizing anterior and posterior approaches. Three levels of anterior cervical discectomy with bilateral uncovertebrectomy was done aiming at decompression and anterior release. Intraoperative, the discs were degenerate with sclerotic bones. Polyetheretherketone (PEEK) cage was later inserted with single screw fixation to prevent anterior dislocation with the idea that the segment was still mobile for further correction posteriorly. The cortical screws were put oblique 30 degrees sagittal, providing buttressing effect at each level (Fig. 2). Posterior instrumentation and fusion were followed to maintain the reduction. Patient subsequently underwent 17 cycles of hyperbaric oxygen therapy. At review one year post-operative, there was clinically good cervical alignment, normal neurology and well healed wounds (Fig. 3). Radiography exhibited a well-maintained reduction and fusion. Histopathology showed bony trabeculae and fatty marrow with cartilaginous tissue.

## Discussion

Osteonecrosis is defined as dead cortical or trabecular bone with empty lacunar spaces^2^. Meanwhile, osteoradionecrosis is a similar condition affecting post-irradiation patients. The complex nature of the disease is believed to be due to metabolic and homeostatic deficiency created by radiation-induced cellular injury with resultant fibrotic, hypo-vascular, and hypo-cellular tissue^3^. The altered blood supply to the area coupled with aberrant condition of the pre-operative malignant lesion will further slow the healing process and impaired bony unions.

The incidence of osteoradionecrosis of sub-axial cervical spine is extremely rare. Khorsandi *et al* reported only twelve patients in his literature review of patients who mainly presented with neck pain. In his personal series, however there were four patients with presentation of three months to eight years post-irradiation and three out of the four patients had higher radiation exposure^1^.

Our patient presented with neck pain and radiculopathy 32 years after the primary surgery due to instability of cervical spine along with impingement of exiting nerve roots. Radiograph upon presentation showed radio-lucent area over C4/C5. In MRI T1WI, the hypo-intense area and hypo-intense on T2WI, non-enhancement post-contrast indicated presence of air within the vertebral body corresponding to intervertebral vacuum cleft sign suggesting osteoradionecrosis^4^. Based on clinical history, MRI findings, and intraoperative findings of sclerotic bone the diagnosis of osteoradionecrosis was made.

We opted for anterior and posterior approach in view of the severe cervical kyphosis, with three levels of discectomy and bilateral uncovertebrectomy for adequate release anteriorly. Anterior surgery was also necessary to prepare a good fusion bed in an irradiated surgical area. Intervertebral cage filled with allograft was finally inserted to maintain the correction achieved. Posteriorly the facet joints were excised at the apex of the deformity and stabilized using cervical pedicle screw construct for superior rigidity and stability. Unique to our technique is that, we utilized a single cortical screw to buttress the cage inserted instead of conventional plating. This was meant to allow us further correction from posterior. We finally managed to restore and maintained the cervical lordosis, and ultimately attained fusion in an irradiated bed.

In an irradiated bed, poor blood supply surrounding the soft tissues and bone make healing process difficult. Hyperbaric oxygen therapy (HBO), provide an excellent adjunct treatment for wound healing as it stimulates the angiogenesis, fibroblast and collagen formation^5^. Our patient was sent for hyperbaric oxygen therapy for 17 cycles, which began at day three post-operatively with the aim of improving wound healing in a scarred and irradiated surgical bed.

## Conclusion

Osteoradionecrosis of the sub-axial cervical spine is rare. Presence of cervical kyphosis makes it a difficult challenge to the surgeon not only to correct kyphosis but also to achieve wound healing, prevent infection and achieve union in an irradiated bed. Anterior cervical discectomy with bilateral uncovertebrectomy provided good decompression, anterior release and bed for fusion with a resultant correction and maintenance of cervical lordosis with resultant of a stable and painless cervical spine. Finally, hyperbaric oxygen therapy provides an excellent adjunct to prevent wound complications.
